# Pigment epithelium derived factor drives melanocyte proliferation and migration in neurofibromatosis café au lait macules

**DOI:** 10.1002/ski2.394

**Published:** 2024-06-24

**Authors:** Charlotte Lovatt, Megan Williams, Alex Gibbs, Abdullahi Mukhtar, Huw J. Morgan, Simone Lanfredini, Carlotta Olivero, Gill Spurlock, Sally Davies, Charlotte Philpott, Hannah Tovell, Peter Turnpenny, Dilair Baban, Sam Knight, Hilde Brems, Julian R. Sampson, Eric Legius, Meena Upadhyaya, Girish K. Patel

**Affiliations:** ^1^ European Cancer Stem Cell Research Institute Cardiff University Cardiff UK; ^2^ Division of Cancer and Genetics Institute of Medical Genetics Cardiff University Cardiff UK; ^3^ Clinical Genetics Royal Devon and Exeter NHS Foundation Trust Exeter UK; ^4^ Wellcome Trust Centre for Human Genetics Oxford UK; ^5^ Department of Human Genetics KU Leuven Leuven Belgium

## Abstract

**Background:**

RASopathies, which include neurofibromatosis type 1 (NF1), are defined by Ras/mitogen‐activated protein kinase (Ras/MAPK) pathway activation. They represent a group of clinically related disorders often characterised by multiple Café au Lait Macules (CALMs).

**Objectives:**

To determine, using in depth transcriptomic analysis of NF1 melanocytes from CALM and unaffected skin, (1) the gene(s) responsible for melanocyte proliferation and migration, and (2) the activated signalling pathway(s) in NF1 melanoma.

**Methods:**

Classical NF1 (*n* = 2, who develop tumours) and 3bp deletion NF1 (p. Met992del, who do not develop tumours) (*n* = 3) patients underwent skin biopsies from CALM and unaffected skin. Melanocytes were isolated and propagated, with five replicates from each tissue sample. DNA and RNA were extracted for mutational analysis and transcriptomic profiling with six replicates per sample. Mechanistic determination was undertaken using melanocyte and melanoma cell lines.

**Results:**

All CALMs in NF1 were associated with biallelic *NF1* loss, resulting in amplification of Ras/MAPK and Wnt pathway signalling. CALMs were also associated with reduced *SERPINF1* gene expression (and pigment epithelium‐derived factor (PEDF) levels, the reciprocal protein), a known downstream target of the master regulator of melanocyte differentiation microphthalmia‐associated transcription factor (MITF), leading to increased melanocyte proliferation, migration and invasion. In classical NF1 and melanoma, but not 3bp deletion NF1, there was also activation of the PI3K/AKT pathway. Pigment epithelium‐derived factor was found to reduce cell proliferation and invasion of NF1 melanoma.

**Conclusions:**

Melanocyte proliferation and migration leading to CALMs in NF1 arises from biallelic *NF1* loss, resulting in RAS/MAPK pathway activation, and reduced expression of the tumour suppressor PEDF. Activation of the PI3K/AKT pathway in classical NF1 and NF1 melanoma may facilitate tumour growth.

## Graphical abstract

We show that loss of Pigment Derived Epithelium Factor regulates melanocyte migration in NF1 café au lait macules. Wherein activation of the PI3K/AKT pathway was only observed in patients with pro‐tumourigenic germline mutations.

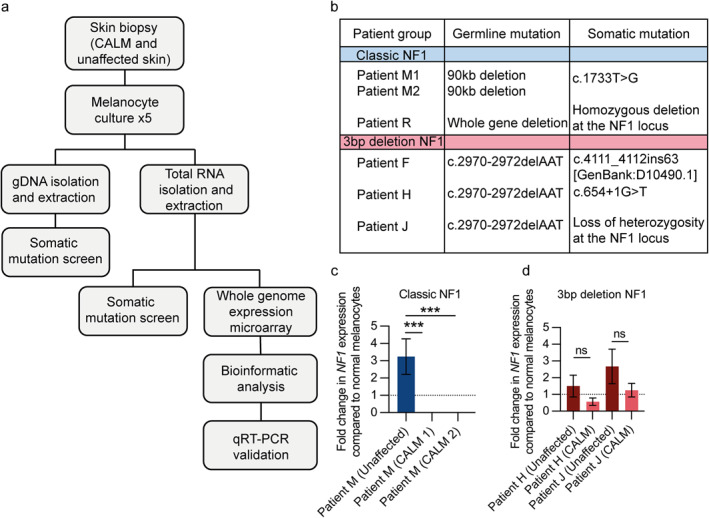

**What is already known?**

- Café au lait maculaes (CALMs) are a hallmark of neurofibromatosis type 1 (NF1), an inherited disease due to mutations in neurofibromin leading to constitutive activation of the Ras/mitogen‐activated protein kinase pathway.
- NF1 patients with a 3bp deletion do not develop tumours.
- NF1 is a common driver mutation in malignant melanoma, which tend to be more invasive and have poorer outcomes.

**What does this study add?**

- NF1 CALMs arise from biallelic loss in melanocytes that acquire a somatic mutation in the second allele, resulting in reduced pigment epithelium‐derived factor (PEDF), which in turn results in melanocyte proliferation and migration.
- NF1 melanoma also demonstrates reduced PEDF, wherein restoration of PEDF can reduce proliferation and invasion.
- NF1 with 3bp deletion does not simultaneously activate the PI3K/AKT pathway, which may explain the absence of tumour formation.

## 1 INTRODUCTION

Café au lait macules (CALMs) are flat, uniformly brown cutaneous lesions that can vary in size, occasionally becoming as large as 20 cm in diameter, which present at birth or within the first few years of life.^1^ While solitary CALMs arise in up to 15% of the population, their presence particularly when multiple, is indicative of a number of genetic disorders that include: neurofibromatosis types 1 (NF1) and 2, Legius syndrome, Bloom syndrome, Cowden disease, Fanconi anaemia, constitutional mismatch repair deficiency syndrome and ataxia telangiectasia.^2^ Among the RASopathies, such as NF1 and Legius syndrome, multiple CALMs are part of the clinical diagnostic criteria.^2^, ^3^ CALMs are associated with an increase in melanocyte number, migration and melanin production, although the cell signalling basis is not known.^4^, ^5^

NF1 is the archetypal RASopathy, a group of inherited syndromes with overlapping phenotypic features caused by germline mutations that lead to activation of the Ras/mitogen‐activated protein kinase (MAPK) pathway.^6^ Other RASopathies include: Legius syndrome, Noonan syndrome, Noonan syndrome with multiple lentigines, capillary malformation–arteriovenous malformation syndrome, Costello syndrome, and cardio‐facio‐cutaneous syndrome. Legius syndrome is caused by germline mutations in *SPRED1*, resulting in CALMs, with or without skinfold freckling; sometimes mild cognitive impairment and attention deficit.^7^, ^8^, ^9^ Classical NF1 is a common autosomal dominant disorder (Von Recklinghausen Disease; OMIM #162200) affecting at least one in 3000 births worldwide.^1^, ^10^, ^11^ It is characterised by findings including CALMs, axillary and inguinal skinfold freckling, neurofibroma, Lisch nodules and skeletal abnormalities. NF1 is caused by germline mutations in the *NF1* gene, with half of all cases resulting from de novo mutations, due in part to the large size (350 kb and 61 exons) of the *NF1* gene.^12^ *NF1* encodes a highly conserved tumour suppressor protein called neurofibromin, which is ubiquitously expressed in many tissues. Neurofibromin negatively regulates Ras by favouring the inactive GDP‐bound form, resulting in inhibition of RAS activity.^13^, ^14^ Of the more than 3197 different constitutional *NF1* mutational variants identified in the Human Gene Mutation Database, very few mutations including those of codons 844–848, 992 and 1809 are associated with defined genotype‐phenotype correlation.^15^ For example, patients with a 3bp in‐frame deletion at c.2970–2972 (p. Met992del, 3bp del NF1) develop CALMS with an absence of neurofibromas and exhibit a lower risk of malignancy.^16^, ^17^, ^18^

Similarly, somatic mutations leading to MAPK pathway activation have been identified as the basis for over 90% of malignant melanoma (MM), notably *BRAF* and *NRAS*.^19^ Specifically, *NF1* and other somatic RASopathy‐associated mutations have been defined as ‘driver’ mutations in 12% of all MM and are present in 45% of MM that are wild type for *BRAF* and *NRAS*.^19^ *NF1* mutations are the main ‘driver’ mutation in amelanotic and desmoplastic MM in which the tumour cells have a spindle cell morphology consistent with a more aggressive phenotype.^20^, ^21^ Furthermore, MM that harbour both *NF1* or *SPRED1* and *BRAF* mutations develop more rapidly and contribute to the frequent relapse during *BRAF* mutation targeted therapies.^22^, ^23^ Thus germline and somatic mutations activating the MAPK pathway alter melanocyte behaviour, giving rise to CALMs and MM respectively. The aim of this study was to compare melanocytes from unaffected skin and CALMs between patients with classical NF1 and 3bp del NF1 to determine through transcriptomic analysis (1) the overlapping basis for CALM formation, and (2) ascertain if differences can elucidate a basis for tumour formation only in classical NF1 and NF1 MM.

## 2 MATERIALS AND METHODS

### 2.1 Patients

**FIGURE 1:**
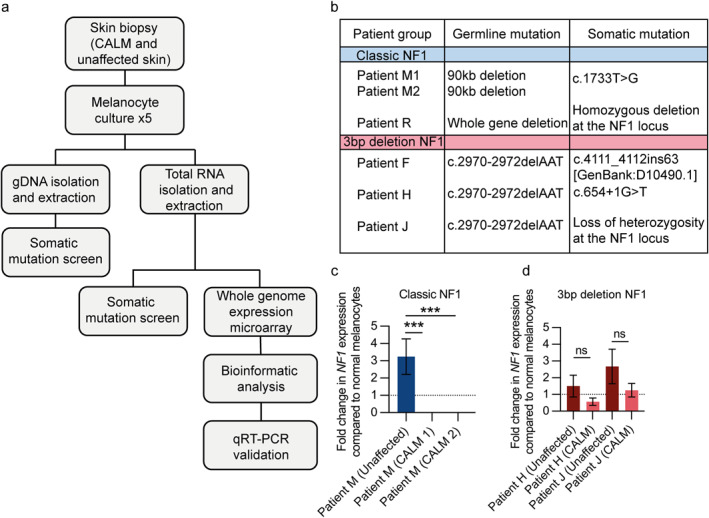
Study design, genotypes and *NF1* expression. (a) Study outline. (b) Somatic mutations within CALMs melanocytes. (c, d) NF1 expression levels within unaffected relative to CALMs melanocytes, 5 replicates per patient sample, relative to normal melanocytes (dotted line). CALMs, café au lait macules; NF1, neurofibromatosis type 1.

This study was approved by National Health Service Research and Development and ethics committees (13/DHD/5637), and also underwent institutional review in KU University, Leuven, Belgium (ML3039). Six patients were included, 2 with NF1 and 3 with p.Met992del‐associated NF1 (clinical and mutation details are shown in Figure 1a). All patients (Figure 1b) underwent 5 mm biopsies, from CALMs and non‐pigmented unaffected skin, for somatic mutation and transcriptomic analysis following melanocyte culture. We were unable to propagate unaffected skin melanocytes from patient F (3bp del NF1).

### 2.2 Somatic mutation screen

Somatic mutations were determined as previously described.^24^, ^25^ Loss of heterozygosity (LOH) was determined using a panel of fluorescently tagged markers encompassing the *NF1* gene region. In addition, samples were screened for (i) microlesions by DNA sequencing (NM_000267) and (ii) intragenic *NF1* deletions/duplications using multiplex ligation‐dependent probe amplification (MLPA). To determine the mutant DNA strand, RNA was reverse transcribed and the cDNA was PCR amplified in 24 overlapping fragments.

### 2.3 Gene expression profiling

RNA from melanocyte cultures of CALM and unaffected skin biopsies were subjected to whole transcriptome expression microarray analysis. RNA from each melanocyte culture was applied to array‐based platforms: Illumina Omni1 Quad, Illumina Human HT12v4.0 Expression BeadChip in Oxford, UK. Fluorescence emissions by Cy3 were imaged using iScan imaging system and GenomeStudio 2011 software (Illumina, San Diego, CA).

### 2.4 Bioinformatics

Raw microarray data as sample probe profiles were imported into R (version 3.6.1) and data was read using lumiR function (detection threshold of ≤0.05). Quality control was then performed through generation of boxplots, density plots, and dendrograms, from which outlier probes were removed and the same plots were re‐generated. Variance stabilisation was then performed using the VST method and data was then normalised using quantile normalisation. Quality control plots were re‐generated for normalised data and assessed before proceeding. Following quality assessment and normalisation, absent probes were removed and then limma model fitting was performed to generate lists of differentially expressed genes (DEGs). These DEGs formed the basis for comparing CALMs melanocytes to unaffected skin melanocytes and between patients leading to the identification of a common pool of DEGs which define archetypal melanocytic change. Unfiltered DEGs with logFC values were imported into R and transposed. A meta‐table was also made. A principal component analysis (PCA) object was created using the prcomp function. The autoplot function (ggplot package) was then used to generate biplots using the metadata information as overlays and points coloured by Sample_ID.

DEGs were also imported into Ingenuity Pathway Analysis (IPA) software (Qiagen) and filtered for adjusted *p* values <0.05. A causal network analysis was performed on the filtered dataset. Comparison analyses were also performed in IPA to generate Venn diagrams. Filtered DEGs were then pre‐ranked from most‐expressed to least‐expressed and imported into Gene Set Enrichment Analysis (GSEA) software (Broad Institute) where they were run against the hallmark (H) gene sets summarising specific well‐defined biology states or processes, and the reactome curated canonical pathways gene set (C2:CP) derived from the reactome pathway database. Normalised enrichment scores (NES) from GSEA were collated and used to generate heatmaps using Morpheus (Broad Institute) where samples and gene sets were hierarchically clustered using Pearson Correlation.

### 2.5 Cell lines

Epidermal melanocytes were expanded for five passages in vitro as previously described.^4^ All cell lines were sub‐cultured using Trypsin‐EDTA solution (Gibco, UK) and propagated in RPMI 1640 (LNCaP, MeWo, M14, Malme‐3M, COLO‐792, SK‐MEL‐30), DMEM (A375), Media 254 (FM291) or MCDB 153 medium (WM3523). Somatic mutations for each of the six melanoma lines (MeWo, M14, A375, Malme‐3M, WM3523, COLO‐792, and SK‐MEL‐30) were obtained from the publicly available cancer knowledgebase canSAR (http://cansar.icr.ac.uk) and confirmed by PCR.

### 2.6 Quantitative real‐time PCR

qRT‐PCR was undertaken using the QuantStudio 7 Flex Real‐Time PCR System (ThermoFisher) to determine fold changes using the following gene probes *NF1* (Hs01035108_m1), *MITF* (Hs01117294_m1), and *SERPINF1* (Hs01106937_m1/Hs01106934_m1). Each well was plated in triplicates with normal primary melanocytes (FM291) used as control and *GAPDH* (Hs02758991_g1) or *β‐Actin* (Hs00357333_g1/Hs01060665_g1) expression as a reference transcript.

### 2.7 Western blot

Cell lysates from cell pellets were prepared in 50 μL RIPA buffer and protein concentrations determined by Pierce BCA assay (ThermoFisher). Proteins were run on 7.5% BIORAD Clarity gel transferred on mini PVDF membranes using the Trans‐Blot Turbo Transfer System and Trans‐Blot Turbo Mini Transfer Packs (Bio‐Rad). Membranes were blocked with 10% bovine serum albumin (BSA, Sigma‐Aldrich) or 10% skimmed milk and probed using the following antibodies: NF1 (H12, Santa Cruz Biotechnology and D7R7D, Cell Signalling Technology), pERK (Erk1/2, Cell Signalling Technology), total ERK (Erk1/2, Cell Signalling Technology), pAKT (D9E, Cell Signalling Technology), total AKT (C67E7, Cell Signalling Technology), GAPDH (MAB374, Cell Signalling Technology), secondary antibodies (ab98693 and ab97051, Abcam). Blots were visualised using Luminate Forte chemiluminescent HRP detection reagent (MerckMilipore) using the BioRad Gel Doc XR^+^ Gel Documentation System.

### 2.8 *NF1* siRNA transfection

Cells were incubated with *NF1* or non‐target siRNA (ON‐TARGETplus siRNA, GE Healthcare Dharmacon) in Lipofecatmine 2000 (Invitrogen) for 24 or 72 h.

### 2.9 *PEDF* vector transduction

Cells were transduced by culturing on top of lentiviral particles, pLV‐IRES‐Venus or pLV‐PEDF‐IRES‐Venus (Cyagen biosciences), bound to retronectin (Clontech) coated 12‐well plates for 1 week before enrichment by flow sorting.

### 2.10 Two chamber culture insert migration assay

Fifty thousand FM291 cells per chamber were cultured with inserts, after 5 h the insert was removed and the chambers imaged every 10 min for 24 h using a Leica DMI6000 B Fluorescent Time Lapse Microscope. Wound area coverage was calculated using the MRI Wound Healing Tool in ImageJ.

### 2.11 Single cell migration

Single cell migration analysis was undertaken on 3000 cells in a 24 well plate, with images taken at 10‐min intervals for 24 h using a Leica DMI6000 B Fluorescent Time Lapse Microscope. CellTracker programme in MATLAB was used to track cells (*n* = 41–123) to give a final average distance moved from origin.

### 2.12 BrdU proliferation assay

Proliferation was quantified using the BrdU Cell Proliferation ELISA Kit (ab126556, abcam) as per manufacturer's instructions.

### 2.13 Transwell invasion assay

Thirty thousand cells were seeded per transwell and cultured for 24 h. The underside of the inserts were stained with Crystal Violet and the intensity of Crystal Violet quantified using ImageJ.

### 2.14 Statistical analysis

Unless otherwise stated, Student's *t*‐test was used to measure significance (*p* ≤ 0.05) between two groups, while one‐way ANOVA was used when comparing multiple groups. All data represents at least three biological replicates.

## 3 RESULTS

### 3.1 Biallelic loss in CALMs

Melanocytes from CALMs from two NF1 patients demonstrated LOH (Figure 1b). Likewise, DNA sequencing identified pathogenic or likely pathogenic somatic mutations affecting the second *NF1* allele in CALMs form all NF1 patients. All mutations were verified by cDNA PCR amplification. As expected, *NF1* gene expression was undetectable in CALMs from classical NF1 patients but present in melanocytes from their unaffected skin (Figure 1c). By contrast, *NF1* expression was detected in both unaffected skin and CALM melanocytes from patients with 3bp deletion NF1 (Figure 1d). These findings suggest that CALM formation is associated with bi‐allelic *NF1* loss.

### 3.2 Ras/MAPK activation in CALMs

**FIGURE 2:**
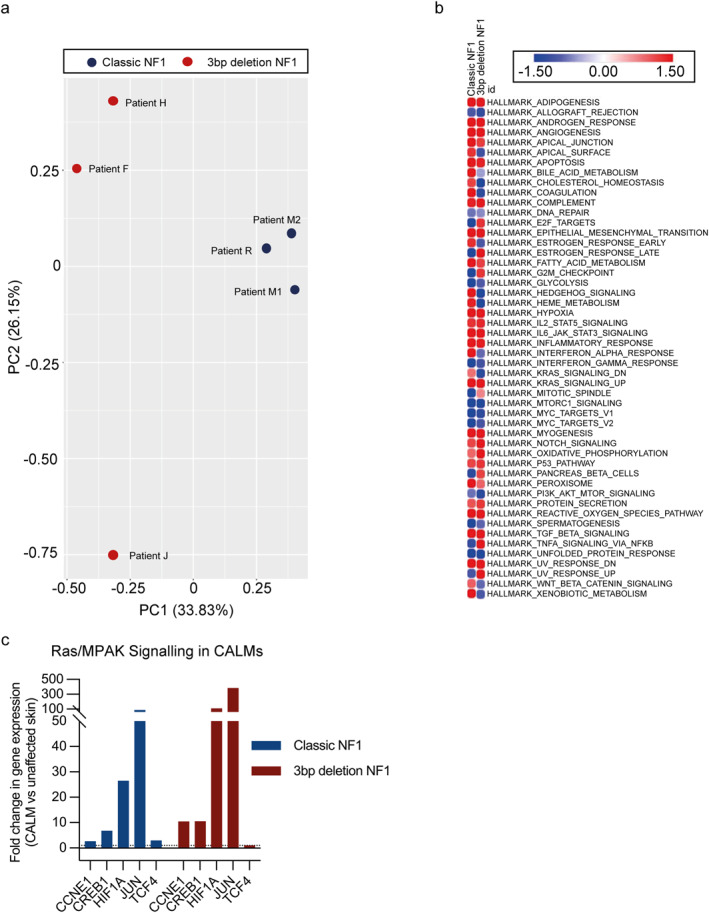
Comparative transcriptomic analysis of Classic neurofibromatosis 1, 3bp deletion neurofibromatosis 1 and Legius syndrome. (a) Principal component analysis plot of individual patient CALMs transcriptional profiles based on genotype. (b) Heatmap showing Gene Set Enrichment Analysis data using Hallmark gene sets of averaged differential expression genes for each genotype. (c) qRT‐PCR comparing CALMs versus unaffected skin melanocyte mRNA of classic and 3bp deletion neurofibromatosis 1 for individual downstream Ras/mitogen‐activated protein kinase regulated genes shown as averages. CALMs, café au lait macules.

**FIGURE 3:**
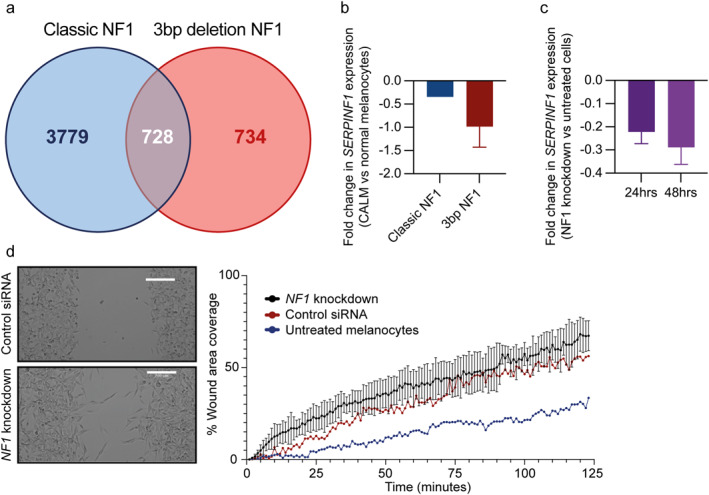
Café au Lait Macules characterised by decreased expression of pigment epithelium‐derived factor. (a) Venn diagram of unfiltered differentially expressed genes shared between the two NF1 genotypes. (b) qRT‐PCR for the PEDF gene *SERPINF1* comparing CALMs mRNA of classic and 3bp deletion neurofibromatosis 1 versus normal melanocytes. (c) qRT‐PCR for the PEDF gene, *SERPINF1*, expression comparing normal melanocytes treated with NF1‐targeting and non‐targeting siRNA. (d) Migration assay comparing normal melanocytes and those treated with NF1‐targeting and non‐targeting siRNA. NF1, neurofibromatosis type 1; PEDF, pigment epithelium‐derived factor.

Bioinformatic transcriptomic analysis showed clustering based on germline genotype with 59.98% separation between classical NF1 and 3bp deletion NF1 by PCA of CALM and unaffected skin melanocytes using unfiltered DEGs (Figure 2a). The absolute threshold was set to 0.5 log2 fold change, to give a +/− 1.4‐fold change, to include the maximum number of DEGs that were significant. Furthermore, this primary segregation could not be explained by *NF1* gene expression levels compared to unaffected skin (Figure 1c,d). We next used IPA™ to undertake causal network and comparison analyses of the DEGs between CALM and unaffected skin melanocytes; which led to the identification of a common pool of 728 genes (Figure 3a, Table S1). 60%, 432 of the common pool genes were recognised as enriched in melanoma (Table S2). Gene set enrichment analysis, using the Hallmark gene sets, demonstrated enrichment for RAS signalling (Figure 2b). Ras/MAPK pathway activation in CALM melanocytes was validated by qRT‐PCR of known Ras/MAPK pathway regulated genes that showed increased expression compared to unaffected skin for classical and 3bp deletion NF1 (Figure 2c). As would be expected, germline mutations in *NF1* were upstream of RAS and therefore showed no selection for specific Ras isoforms, bioinformatic analysis showed no specific enrichment of target gene sets for HRAS, KRAS or NRAS (Figure S1). Thus, classical NF1 and 3bp del NF1, despite differences in germline mutation and transcription signatures, result in Ras/MAPK pathway activation. Further analysis of the overlapping 728 genes showed 579 genes were concordantly regulated, 10 up‐regulated and 569 down‐regulated with ≥0.5 log fold change.

### 3.3 PEDF is a regulatory node in NF1

Ingenuity Pathway Analysis comparison analyses of the DEGs between CALM and normal melanocytes identified pigment epithelium‐derived factor (PEDF and *SERPINF1*, protein and gene names respectively) as a key regulatory node in CALMs in classic and 3bp deletion NF1, when compared to normal melanocytes (Table S3). *SERPINF1*, which is a target gene for the master regulator of melanocyte differentiation microphthalmia‐associated transcription factor (MITF), was consistently under‐expressed in all CALMs samples compared to normal melanocytes, which we confirmed by qRT‐PCR (Figure 3b). Since reduced *SERPINF1* expression represented a potential common transcriptional node in CALMS, we next sought to determine whether loss of neurofibromin was sufficient to affect *SERPINF1* expression. Using siRNA targeting the *NF1* gene and vector control we observed a reduction in *SERPINF1* relative to vector control (Figure 3c), therefore the loss of NF1 was sufficient to reduce *SERPINF1* expression. To determine the functional consequence of this reduction in *SERPINF1* expression, we assessed cell proliferation and migration both of which are involved in CALM formation. Although there was no change in melanocyte proliferation (data not shown), reduction in *SERPINF1* expression by siRNA *NF1* gene knockdown led to a 10‐fold increase in migration compared to untreated melanocytes; but did not reach significance compared to vector control (Figure 3d). Thus, simultaneous loss of NF1 and PEDF expression in melanocytes increases their migration and may therefore contribute to CALM formation.

**FIGURE 4:**
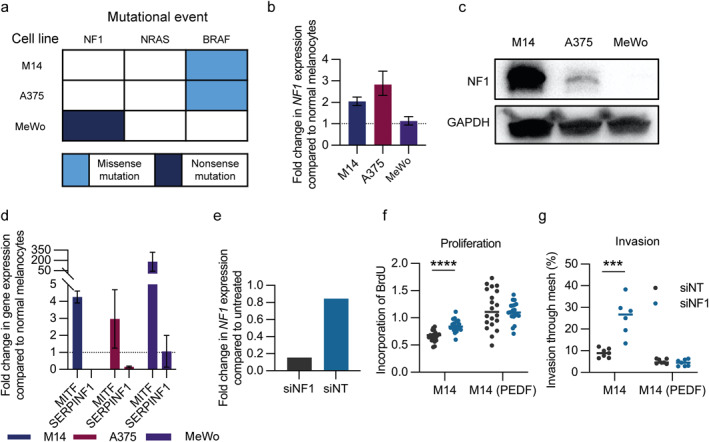
PEDF rescues loss of NF1 associated proliferation and migration. (a) Characterisation of MM cell lines. qRT‐PCR (b) and Western blot analysis (c) for NF1 expression in MM cell lines. (d) qRT‐PCR for MITF and PEDF gene expression in MM cell lines. (e) Validation of NF1‐targeting siRNA treatment of M14 melanoma cells by qRT‐PCR. Functional assessments of M14 melanoma cell proliferation (f) and invasion (g) after NF1 knockdown and PEDF rescue. MM, malignant melanoma; NF1, neurofibromatosis type 1; PEDF, pigment epithelium‐derived factor.

We next examined the functional role of PEDF directly, particularly in the context of melanoma wherein NF1 mutations are associated with more invasive disease in both BRAF mutant and wild type disease. If indeed PEDF represents an important regulatory node in the RASopathies, the effects of neurofibromin loss should be mitigated by PEDF restoration, and thus represent a potential adjuvant therapeutic strategy for NF1 melanoma. We first screened melanoma cell lines for known NF1 mutations (Figure 4a), which we confirmed by qRT‐PCR (Figure 4b) and Western blot analysis (Figure 4c). As expected, the MeWo cell line due to an NF1 nonsense mutation had greater *MITF* expression, consistent with RAS/MAPK signalling, but did not result in an increase in *SERPINF1* expression (Figure 4d). Of the two BRAF mutant cell lines, M14 and A375, we chose the M14 cell line, which already had lower *NF1* and *SERPINF1* levels. M14 siRNA *NF1* gene knockdown led to a 4‐fold reduction in *NF1* expression compared to vector control (Figure 4e), as expected *NF1* knockdown led to a further increase in MAPK pathway activation (data not shown). In contrast to normal melanocytes, loss of *NF1* expression led to increased cell proliferation (*p* < 0.01, Figure 4f), which was not evident in M14 cells transduced with a lentiviral vector to over‐express *SERPINF1*. As expected, loss of *NF1* expression was associated with a significant increase in melanocyte invasion (*p* < 0.01, Figure 4g), however this was blocked by PEDF. Hence PEDF represents an important regulatory node, which in NF1 promotes melanocyte migration and invasion.

### 3.4 PI3K‐AKT pathway activation in classic NF1

We next turned our attention to whether the transcriptomic dataset could provide the basis for tumourigenesis in classical NF1 but not in 3bp deletion NF1, as we had already shown that CALM melanocytes exhibited biallelic loss similar to that observed in tumours arising within RASopathies. We observed activation of the Ras/MAPK pathway (Figure S2a) but also the Wnt pathway in all three studied genotypes (Figure S2b). However, Reactome gene set enrichment identified activation of the PI3K/AKT pathway in only classical NF1 CALM melanocytes, whereas it was downregulated in 3bp deletion NF1 (Figure S2a). Compared to normal melanocytes, there was much greater expression of PI3K/AKT pathway downstream regulated genes in unaffected skin melanocytes from classic NF1 patients when compared to those from patients with 3bp deletion NF1 (Figure S2c). Also, in comparison to normal melanocytes, activation of PI3K/AKT pathway was greater in both unaffected skin and CALM melanocytes in classic NF1 (Figure S2d). Within melanocytes, concordant Ras/MAPK, PI3K/AKT and Wnt pathways are required for activation of MITF for melanocyte development, function, and survival. As expected, MITF‐regulated genes demonstrated enrichment in classic NF1 and less so in 3bp deletion NF1 (Figure S2e). Thus, PI3K/AKT pathway activation may represent an important difference between classic NF1 and the other genotypes, accounting for the increased risk for tumourigenesis in classic NF1.

## 4 DISCUSSION

Genome‐wide transcriptomic microarray analysis has been used in rare pigmentary skin disorders, allowing for comparison between normal melanocytes and those from the affected and unaffected patient skin, in small numbers of patients.^26^ Although classical NF1 is a common disorder there are small numbers of patients with 3bp deletion NF1. To overcome the obvious limitations of a small number of patients in this study, we have undertaken multiple replicates both in culture and subsequent transcriptomic analysis. Germline loss of a single *NF1* allele slightly increases the level of RAS activity^27^ since neurofibromin function is not completely lost in *NF1* heterozygous cells.^28^, ^29^ For CALMs to develop in NF1 patients, the affected melanocytes demonstrate biallelic *NF1* loss‐of‐function mutations, resulting from a second somatic mutation. While we have only examined 5 patients, these findings support earlier studies documenting LOH in CALM lesions and other tumours in NF1 patients.^30^ While in the classic neurofibromatosis phenotype biallelic *NF1* mutations in CALM lesions were associated with a complete loss of *NF1* expression, in the two patients with 3bp deletion NF1 (for whom we had unaffected skin samples for comparison) there was a commensurate reduction in *NF1* expression.

CALM melanocyte Ras/MAPK pathway activation led to an increase in *MITF* and MITF‐regulated genes.^31^, ^32^ We identified 728 differentially regulated genes that represent the CALM transcriptional signature, among which we identified *SERPINF1* as being consistently downregulated. We confirmed that *NF1* knockdown led to reduced *SERPINF1* levels in melanocytes. However, this study did not address a direct link between reduced expression of *NF1 and SERPINF1*; in the context of MM, *SERPINF1* is also a direct target of the tumour suppressor p53.^33^ Several studies have shown an inverse correlation between PEDF expression levels and the progression and metastatic potential in a variety of cancers including ovarian, kidney, hepatocellular, lung and melanoma.^32^, ^34^ A negative correlation between MITF and PEDF expression is associated with the malignant progression of MM.^35^ We confirmed a reduction in expression in NF1 CALMs and MM, and herein show restoring PEDF expression is sufficient to reduce proliferation and invasion.

While all CALM melanocytes exhibited Ras/MAPK signalling pathway activation, increased activity of the PI3/AKT signalling cascade was seen only in melanocytes from patients with classical NF1, both in the unaffected skin but to a much greater extent in CALMs. Activation of the PI3K‐AKT pathway in classical NF1 may therefore account for tumour formation, as the pathway is activated in other tumour forming syndromes: neurofibromatosis 2, Cowden, CLOVES and tuberous sclerosis.^36^ NF1 MM also show an increase in the expression of PI3K and AKT, whose expression has been shown to steadily increase during progression of MM to metastatic.^31^

In summary, CALMs are associated with a discordant loss of PEDF. NF1‐associated tumourigenesis is associated with activation of PI3K/AKT signalling, which is absent in 3bp deletion NF1.

## CONFLICT OF INTEREST STATEMENT

The authors state no conflict of interest.

## AUTHOR CONTRIBUTIONS

**Charlotte Lovatt**: Data curation (equal); formal analysis (equal); investigation (lead); writing – review & editing (equal). **Megan Williams**: Data curation (equal); formal analysis (equal); investigation (lead); writing – review & editing (equal). **Alex Gibbs**: Formal analysis (equal); investigation (equal); writing – review & editing (equal). **Abdullahi Mukhtar**: Formal analysis (equal); investigation (equal); writing – review & editing (equal). **Huw J. Morgan**: Formal analysis (equal); investigation (equal); writing – review & editing (equal). **Simone Lanfredini**: Formal analysis (equal); investigation (equal). **Carlotta Olivero**: Formal analysis (equal); investigation (equal). **Gill Spurlock**: Formal analysis (equal); investigation (equal). **Sally Davies**: Resources (lead). **Charlotte Philpott**: Data curation (equal); formal analysis (equal); investigation (equal). **Hannah Tovell**: Data curation (equal); formal analysis (equal); investigation (equal). **Peter Turnpenny**: Resources (lead). **Dilair Baban**: Conceptualization (equal); investigation (equal). **Sam Knight**: Conceptualization (equal); investigation (equal). **Hilde Brems**: Investigation (equal). **Julian R. Sampson**: Resources (lead). **Eric Legius**: Resources (lead). **Meena Upadhyaya**: Conceptualization (lead); formal analysis (equal); resources (lead); supervision (equal); writing – original draft (equal); writing – review & editing (equal). **Girish K. Patel**: Conceptualization (lead); formal analysis (equal); resources (lead); supervision (lead); writing – original draft (lead); writing – review & editing (lead).

## ETHICS STATEMENT

Not applicable.

## Supporting information

Figure S1

Figure S2

Table S1

Table S2

Table S3

## Data Availability

Data and reagents used in this paper will be made available upon reasonable request to the corresponding author.
