# Supplementary figures and images for: Pigment epithelium derived factor drives melanocyte proliferation and migration in neurofibromatosis café au lait macules
Source: Skin Health Dis. 2024 Jun 24;4(5):e394. doi: 10.1002/ski2.394 (PMC11442068; doi:10.1002/ski2.394)

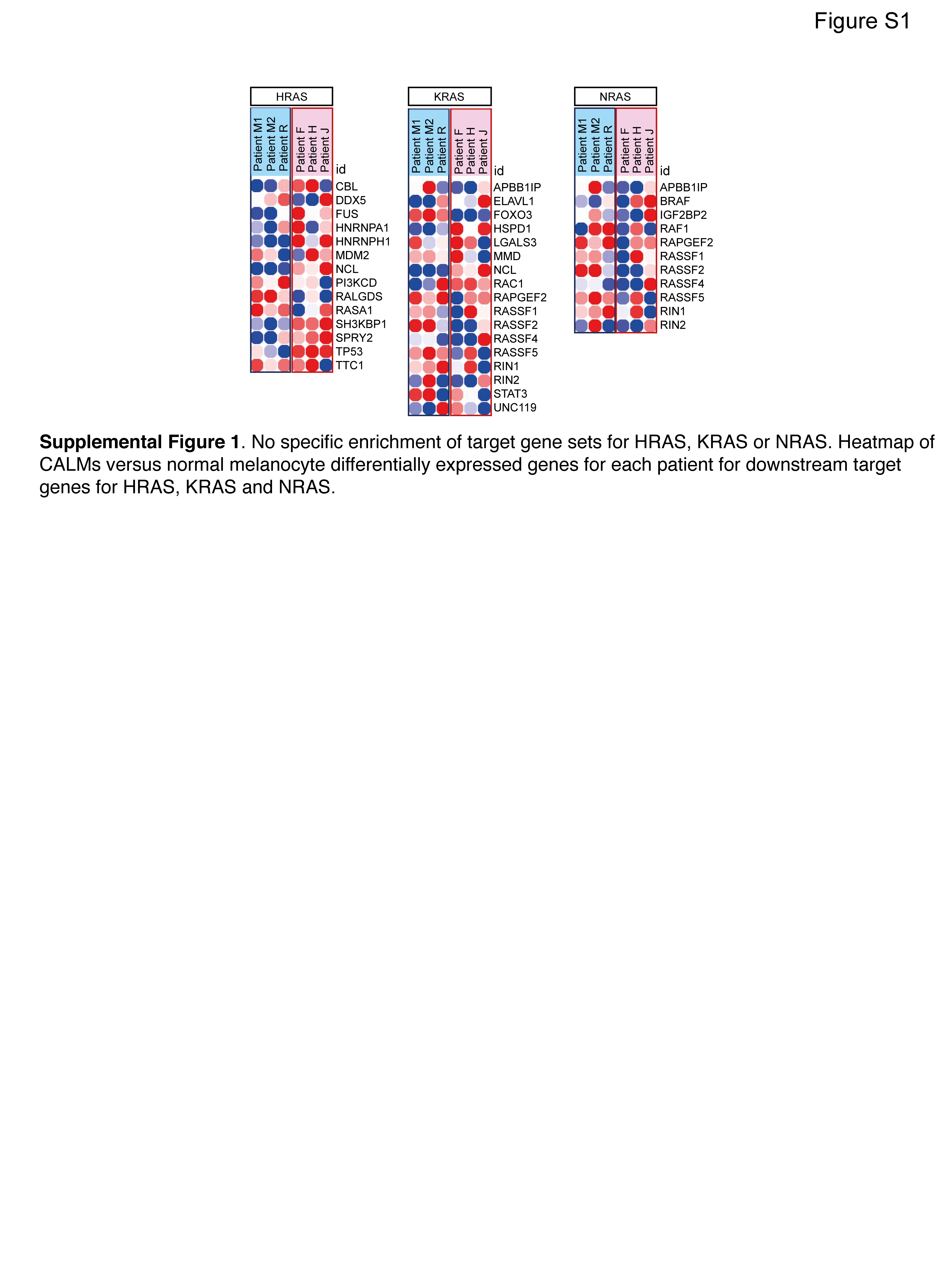

Supplement: Supplementary file 1 — Figure S1 [file SKI2-4-e394-s003.tif]

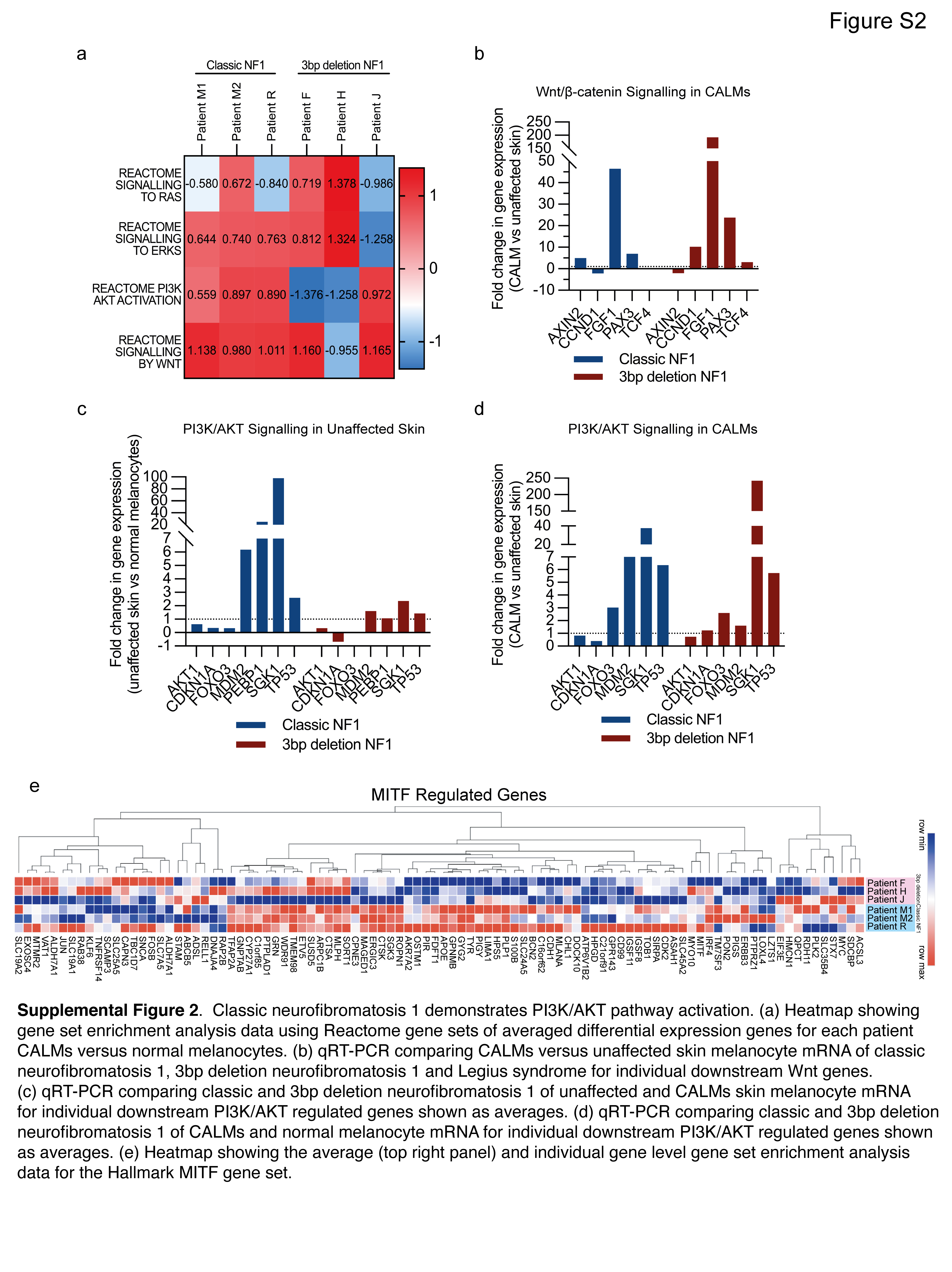

Supplement: Supplementary file 2 — Figure S2 [file SKI2-4-e394-s001.tif]
